# Leveraging convolutional neural networks and hashing techniques for the secure classification of monkeypox disease

**DOI:** 10.1038/s41598-024-75030-y

**Published:** 2024-11-04

**Authors:** Essam Abdellatef, Alshimaa H. Ismail, M. I. Fath Allah, Wafaa A. Shalaby

**Affiliations:** 1 Department of Electrical Engineering, Faculty of Engineering, Sinai University, El-Arish, 45511 Egypt; 2 Information Technology Department, Faculty of Computer and Informatics, Tanta University, Tanta, 31527 Egypt; 3 Department of Electrical Engineering, Faculty of Engineering, Suez University, Suez, 43533 Egypt; 4 Department of Electronic and Electrical Communication Engineering, Faculty of Electronic Engineering, Menoufia University, Menouf, Egypt

**Keywords:** Monkeypox, Cancelable techniques, CNN, And DarkNet-53, Drug discovery, Diseases, Health care, Health occupations, Medical research

## Abstract

The World Health Organization declared a state of emergency in 2022 because of monkeypox. This disease has raised international concern as it has spread beyond Africa, where it is endemic. The global community has shown attention and solidarity in combating this disease as its daily increase becomes evident. Various skin symptoms appear in people infected with this disease, which can spread easily, especially in a polluted environment. It is difficult to diagnose monkeypox in its early stages because of its similarity with the symptoms of other diseases such as chicken pox and measles. Recently, computer-aided classification methods such as deep learning and machine learning within artificial intelligence have been employed to detect various diseases, including COVID-19, tumor cells, and Monkeypox, in a short period and with high accuracy. In this study, we propose the CanDark model, an end-to-end deep-learning model that incorporates cancelable biometrics for diagnosing Monkeypox. CanDark stands for cancelable DarkNet-53, which means that DarkNet-53 CNN is utilized for extracting deep features from Monkeypox skin images. Then a cancelable method is applied to these features to protect patient information. Various cancelable techniques have been evaluated, such as bio-hashing, multilayer perceptron (MLP) hashing, index-of-maximum Gaussian random projection-based hashing (IoM-GRP), and index-of-maximum uniformly random permutation-based hashing (IoM-URP). The proposed approach’s performance is evaluated using various assessment issues such as accuracy, specificity, precision, recall, and fscore. Using the IoM-URP, the CanDark model is superior to other state-of-the-art Monkeypox diagnostic techniques. The proposed framework achieved an accuracy of 98.81%, a specificity of 98.73%, a precision of 98.9%, a recall of 97.02%, and f_score_ of 97.95%.

## Introduction

The Monkeypox virus is a smallpox virus, an infectious disease related to the zoonotic virus Orthpoxvirus, which is related to cowpox^1^. Monkeypox cases have been increasing in Africa and other countries^2^. This disease is transmitted by rodents and monkeys and easily spreads from one human to another during body contact. In 1958, the first discovery of this disease by Preben Alexander in monkeys was in a laboratory in Denmark^3^. The first detection of this disease in humans was recorded in 1970 in the Congo^4^. Subsequently, the virus spread to other countries, such as Liberia, Sierra Leone, and Nigeria^5^. In 2003, the first case of Monkeypox appeared in the United States and was the result of contact with prairie dogs that were sick without being reported. From 2017 to 2019, the disease began to spread in Nigeria, with 122 deaths reaching 7 cases^6^. To date, the number of Monkeypox cases has reached 90,000, and 115 deaths have been reported. It has spread widely to countries outside Africa, such as the United States of America, Germany, France, Australia, and Sweden^7–16^.

The main symptoms that appear early in cases influenced by this disease are headache, fever, chills, muscle pain, fatigue, and skin rashes, such as chickenpox^17^. Monkeypox is considered a viral disease similar to coronavirus, but it is less widespread. Despite this, Monkeypox began to spread in Africa in 1990, until cases increased and began to spread until the number of cases reached 5,000 in 2020, after which it began to spread in the United States of America and Europe^18^. According to the Centers for Disease Prevention, the number of cases reached approximately 83,424 by 2022. There is still no cure for the Monkeypox virus, according to what was praised by the National Center for Disease Eradication. However, vaccination is carried out using two drugs, ticovirimat and brincidofovir, which are used to treat the smallpox virus, according to what was provided by the Center for Disease Control (CDC)^19^. This vaccination has become a solution to prevent the Monkeypox virus in many countries, but the United States of America no longer gives it to humans^20^. Procedures for diagnosing Monkeypox include initial observations of the unusual characteristics of existing skin lesions and a history of current exposure. This virus can be diagnosed using an electron microscope to test for skin diseases, and it can also be diagnosed using PCR, as it has also been used to diagnose COVID-19 patients^21^. As of September 27, 2023, approximately 90,630 cases of Monkeypox had been confirmed in laboratories, with 115 deaths recorded. The following figure shows the confirmed cases of the top 15 countries in terms of the number of confirmed infections, as the United States of America is considered the highest country, with the rate of infections reaching 30,861 and deaths 54, followed by Brazil, where the number of infections reached 10,967 and deaths 16. Furthermore, the number of confirmed cases in Spain reached 7,850, with three deaths. Colombia, France, Germany, and Mexico have approximately 4000 cases of this disease, while Argentina, China, Chile, Canada, and Portugal have around 1000 cases^19^.

Deep learning (DL) is a subdivision of machine learning (ML) that takes cues from the operation of the human brain. It is used to extract and categorize characteristics in images. The primary advantage of this technique is its use in unsupervised learning, where learning is done from unlabeled data examples. Due to its ability to consume unlabeled data, operate without feature engineering, and make highly accurate and precise predictions, machine learning has become a widely used technology in industries such as self-driving cars, face recognition, object detection, and image classification. Recent research has proven that computer vision, machine learning, and deep learning can be utilized to automate the identification of various illnesses in the human body^22^. Feature extraction stage is conducted using a convolutional neural network (CNN), which is a subset of deep learning. It is in charge of acquiring an input image and allocating learn-able weights as well as biases to different objects in that image. It utilizes appropriate filters to extract temporal and spatial dependencies within an image^23^. Each layer in the CNN carries out a distinct operation on the input images. A variety of innovations has led to a notable enhancement in the representational capacity of CNN. Notably, there has been significant attention given to the idea of leveraging the information, depth, and width of the architecture. Similarly, the concept of utilizing a cluster of layers as a structural component is becoming increasingly popular. A Convolutional Neural Network (CNN) is composed of various layers, each with a distinct function in processing input images. The key layers that contribute to constructing CNN architectures include convolutional, pooling, batch normalization, depth concatenation, and fully connected layers. The main contributions of this study are as follows:

1. Providing a secure Monkeypox classification system to sufficiently diagnosis, Monkeypox cases and impose complete confidentiality about patient information.
2. Extraction of appropriate and distinctive features from Monkeypox skin images using a highly efficient pre-trained CNN (DarkNet-53).
3. Preserving patient information using cancelable techniques.

In the next sections, we discuss the related work in Sect. 2, the architecture of the proposed CanDark model in Sect. 3, the experimental results in Sect. 4. Finally, the paper is concluded in Sect. 5.

## Related work

There are various advanced techniques for identifying and categorizing illnesses through deep learning models, including the identification of COVID-19. Ozkaya et al.^24^ proposed a hybrid model for classification that employed an SVM classifier utilizing deep feature extraction approaches such as Resnet-50, GoogleNet, and VGG-16. Cohen et al.^25^ established a strategy for predicting the severity score of COVID-19 pneumonia using X-ray pictures. Various pre-trained networks were utilized to categorize Monkeypox infections in^26,27^. More than a million images were used to train the pre-trained networks. The task they have is to categorize input images into 1000 or more element classes, such as pencil, coffee mug, keyboard, and many others. The utilization of these networks, in addition to transfer learning, provides easier and faster performance than the training of the model from scratch. The concept of deep transfer learning depends on using these pre-trained networks to classify new input images^28,29^. Table 1 provides previous and relevant research on various Monkeypox classification approaches.

**Table 1:** Previous and relevant research.

Method	Dataset	Classes	Results
Sahin (2022)^30^	228 images (MSLD)	Binary	The accuracy of ResNet18 = 86.8%, GoogleNet = 82.22%, EfficientNetbo = 91.11%, NasnetMobile = 86.6%, ShuffleNet = 80%, MobileNetv2 = 91.11%
Irmak (2022)^31^	770 images in MSLD	Multiclass	Accuracy = 91.38% Precision = 90% Recall = 86% F_score_ = 88%
Alcala (2023)^32^	2067images of (MSLD)	Binary	Accuracy = 97% AUC = 0.76 Loss function = 0.1442
Akin (2022)^33^	572 images in Monkeypox Skin Images Dataset (MSID)	Binary	Accuracy = 98.25%, Sensitivity = 96% F_score_ = 98%
Sitaula (2022)^34^	1753 images in Monkeypox dataset	Multiclass	Precision = 85% Recall = 85% F_score_ = 85% Accuracy = 87.13%
Ali (2022)^35^	(MSLD) which was developed by the authors.	Binary Monkeypox and Others (Chickenpox, Measles).	The accuracy of ResNet50 = 82.96 (± 4.57%), VGG16 = 81.48 (± 6.87%).
Altun (2023)^36^	2056 images with augmentation of Monkeypox Skin Lesion Dataset.	Two different classes (positive and negative)	F_score_ = 98% Accuracy = 96% Recall = 97%
Islam (2022)^37^	Monkeypox Skin Image Dataset 2022	Binary	ShuffleNet-V2 model has achieved the best results of 79% accuracy with 85% precision.
Haque (2022)^38^	572 images of MSID	Binary	Xception-CBAM-Dense accuracy = 83.89%
Ahsan (2022)^39^	90 images for study one and 1754 images for study two of “Monkeypox 2022” Which was collected by the authors	(Chickenpox, Measles, and Normal images) images	(study one) Accuracy = 97 ± 1.8% AUC = 0.97 (study two) Accuracy = 88 ± 0.8% AUC = 0.867

## The proposed Framework

**Fig. 1:**
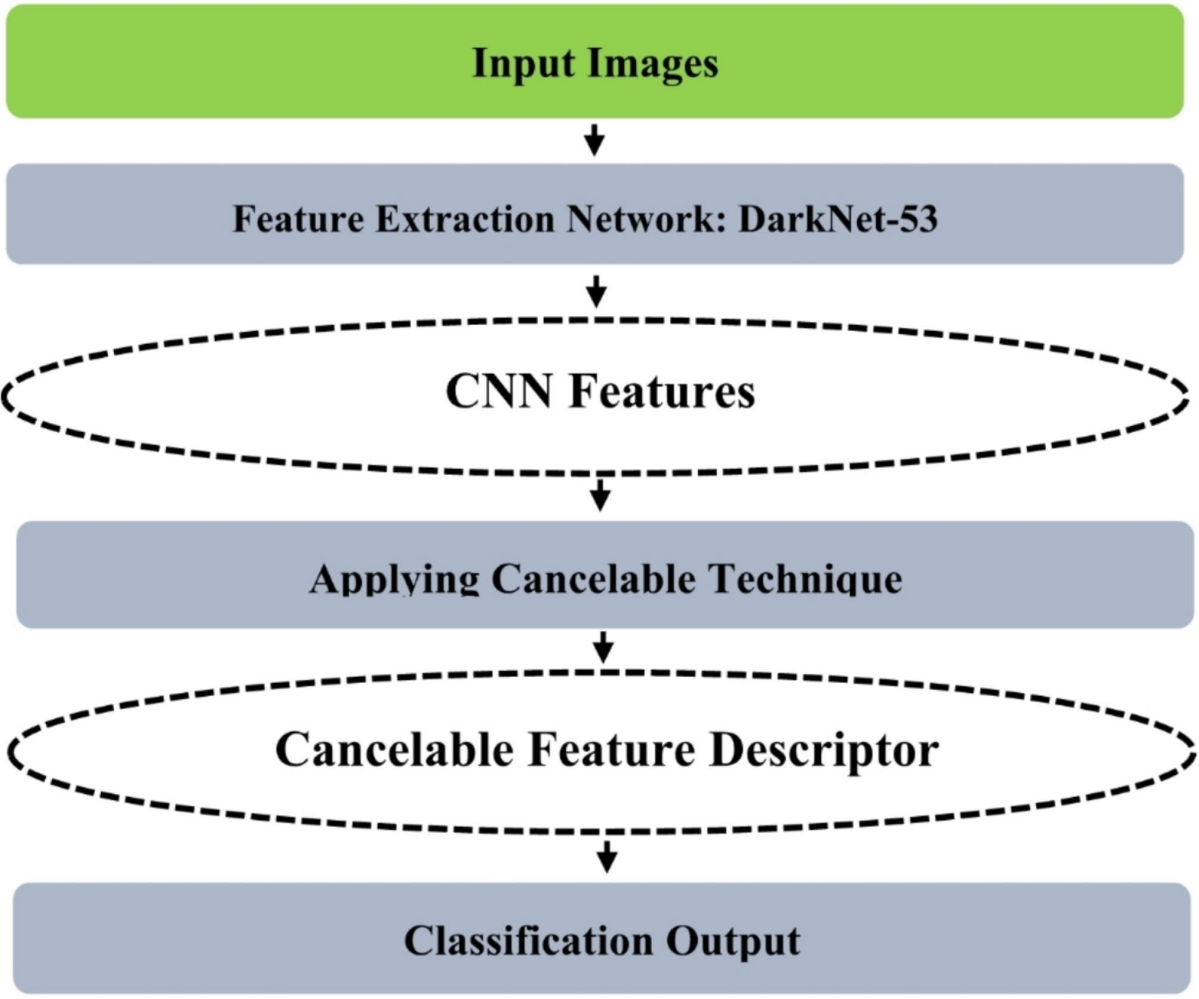
The proposed CanDark model.

To preserve patient information and impose complete confidentiality, we need a secure classification system. The proposed approach is a secure machine-learning-based system for classifying various cases of Monkeypox disease (Fig. 1). The proposed approach incorporates machine learning to extract sufficient, appropriate, and distinctive features from input images. The DarkNet-53 CNN model is developed and used for feature extraction. To protect patient information, various cancelable techniques are evaluated: Bio-Hashing, MLP Hashing, IoM-GRP, and IoM-URP. Cancelable methods are applied to the extracted features before the classification stage. Cancelable methods are preferred to other encryption techniques as they provide efficient protection of information and, at the same time, do not affect the classification accuracy of the system. All procedures performed in studies involving human participants were in accordance with the ethical standards of the institutional and/or national research committee and with the 1964 Helsinki declaration and its later amendments or comparable ethical standards.

### Dataset

**Fig. 2:**
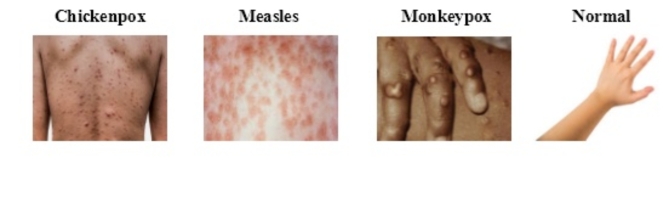
Sample of images of the classes (Chickenpox, Measles, Monkeypox, and Normal) of the Monkeypox Skin Image Dataset.

The Monkeypox Skin Image Dataset is used, which contains 770 images classified into four categories: (107) chickenpox cases, (91) measles cases, (279) cases of Monkeypox (279), and (293) normal cases^40^. Classes are divided into 70% for training and 30% for testing. Table 2 describes the Monkeypox Skin Image Dataset. Furthermore, Fig. 2 provides a sample of images for all classes.

**Table 2:** Number of images, training set, and testing set for the classes (Chickenpox, Measles, Monkeypox, and normal) of the Monkeypox skin image dataset.

Class	#Images	Training set	Testing set
Chickenpox	107	74	33
Measles	91	64	27
Monkeypox	279	196	83
Normal	293	205	88

### Feature extraction network: DarkNet-53

DarkNet-53 is a convolutional neural network that extracts features from images. This network consists of 53 deep convolution layers followed by a batch normalization (BN) layer and a Leaky ReLU layer which represents the activation function. Leaky ReLU layer could be described as the following: $f\left(x\right)=x\; {for}\; x\ge 0\; {and}\; f\left(x\right)=scale\text{*}x\; for\; x<0.$ Leaky ReLU layer provides a small slope for negative values. Furthermore, DarkNet-53 is the backbone of the YOLOv3 object detection methodology. The architecture of the DarkNet-53 is described in details in Table 3.

**Table 3:** Architecture of feature extraction network (DarkNet-53).

	Type	Filters	Size	Output
	Convolutional	32	3 × 3	256 × 256
	Convolutional	64	3 × 3 /2	128 × 128
1×	Convolutional	32	1 × 1	128 × 128
	Convolutional	64	3 × 3	
	Residual			
	Convolutional	128	3 × 3 /2	64 × 64
2×	Convolutional	64	1 × 1	64 × 64
	Convolutional	128	3 × 3	
	Residual			
	Convolutional	256	3 × 3 /2	32 × 32
8×	Convolutional	128	1 × 1	32 × 32
	Convolutional	256	3 × 3	
	Residual			
	Convolutional	512	3 × 3 /2	16 × 16
8×	Convolutional	256	1 × 1	16 × 16
	Convolutional	512	3 × 3	
	Residual			
	Convolutional	1024	3 × 3 /2	8 × 8
4×	Convolutional	512	1 × 1	8 × 8
	Convolutional	1024	3 × 3	
	Residual			
	Average Pooling			
	Fully Connected	1000		
	Softmax	-		

### Cancelable biometric techniques

Cancelable biometrics is the best protection system from the point of view of researchers and is now used to enhance template security and privacy protection in biometric systems^41^. Protection is achieved by converting the original properties into another property with a new representation that is not reversible using certain transformations. The new form after the conversion is called a revocable biometric template. A cancelable biometric must possess four characteristics: irreversibility, diversity, reversibility, and reusability^42^. There are several cancelable biometric techniques, and we concentrate on image-hashing techniques. One of the basic functions of encryption is a hash function. This is called the compression or contraction function and can be used in passwords and file authentication. The hash takes a number of bits from the input and outputs, and 128 bits is a fixed number because it is impossible to recover the original from the output^43^. Password authentication requires a comparison of the stored copies of the hash with the entered password hash.

#### Bio-hashing

**Fig. 3:**
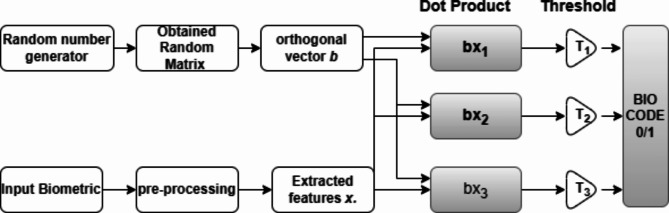
Bio-hashing representation.

Bio-Hashing is considered as a feature extraction technique that uses wavelet transform to extract biometric features^44^. The features *x*, $x={R}^{N}$ are extracted from input data for *N* bits. Also, the orthogonal random vector is defined as $b={R}^{N}.$ The dot product of the feature vector and the random vector is calculated to represent the Bio-Hash *C* as in the following equation:

1 $$C=sig\left(\sum_{i}x{b}_{i}-\varOmega\right)$$

where *sig* is denoted as the signum function and $\varOmega$ is the threshold. Additionally, the operation of Bio-Hashing technique is described through the following diagram in Fig. 3.

#### Multi-layer perceptron (MLP) hashing

**Fig. 4:**
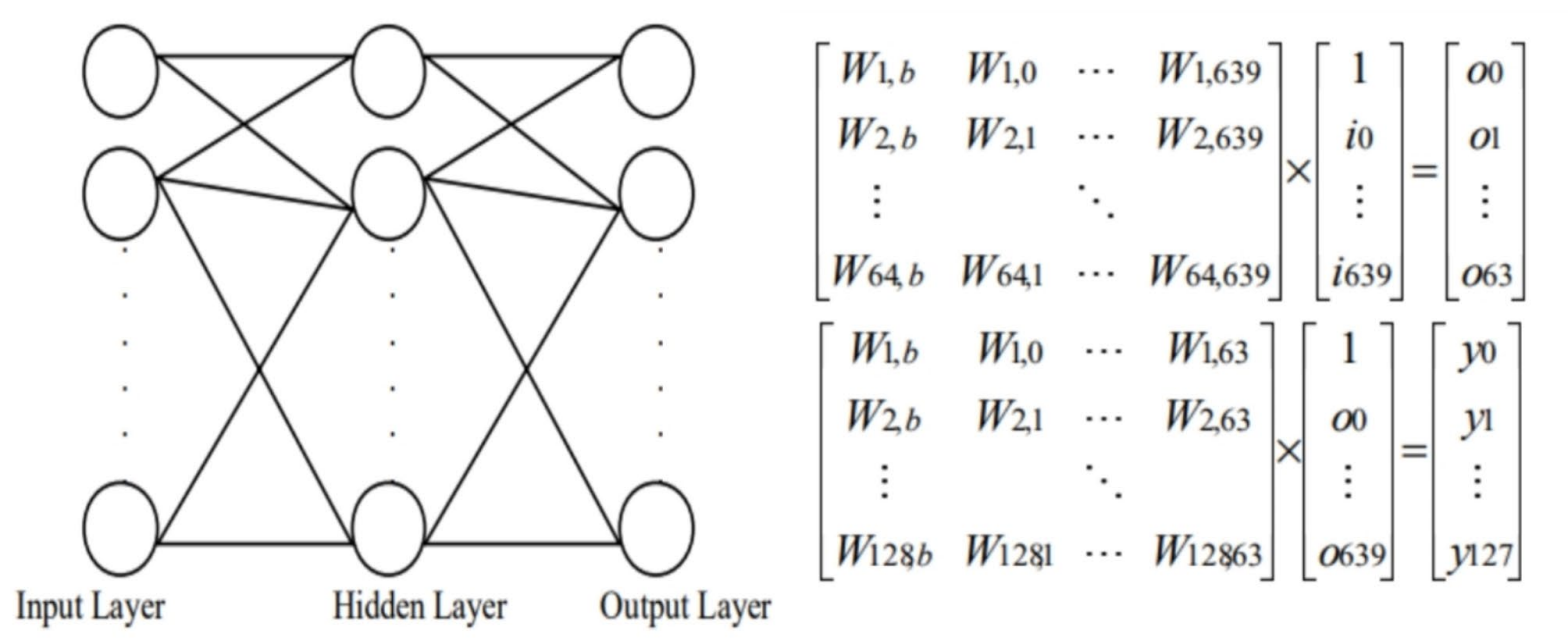
MLP layers and representation in a matrix.

Using MLP hashing offers many useful attributes. Changing the values of the initial weights of the network leads to a complete difference in the segmentation output. Also, the construction of the network can be easily modified by adding new neurons to generate many output bits. MLP network is used as one-way hashing. MLP Network consists of several layers of neurons that called input (*i*), hidden (*o*) and output (*y*) layers as described in Fig. 4. The output layers are from *y*_*0*_ to *y*_*127*_, *O*_*0*_ to *O*_*63*_ are the output of the hidden layers, and the weights are denoted as *Wx*,* y*^45^. The sigmoidal function is used for finding the *o* and *I*. The range of the output is between 0 and 1.

#### Index-of-maximum Gaussian random projection-based hashing (IoM-GRP)

**Fig. 5:**
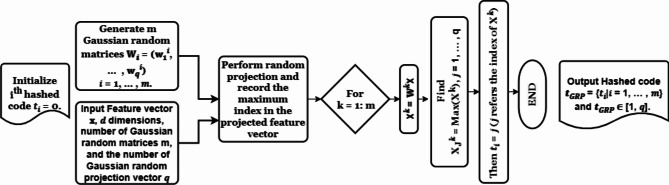
IoM-GRP algorithm.

The IoM hashing type converts the biometric feature vector to maximum ranking hashing code. IoM-GRP is derived from IoM hashing and depends on Gaussian random projection vector^46^. IoM-GRP could be summarized in the following diagram in Fig. 5.

#### Index-of-maximum uniformly random permutation-based hashing, (IoM-URP)

**Fig. 6:**
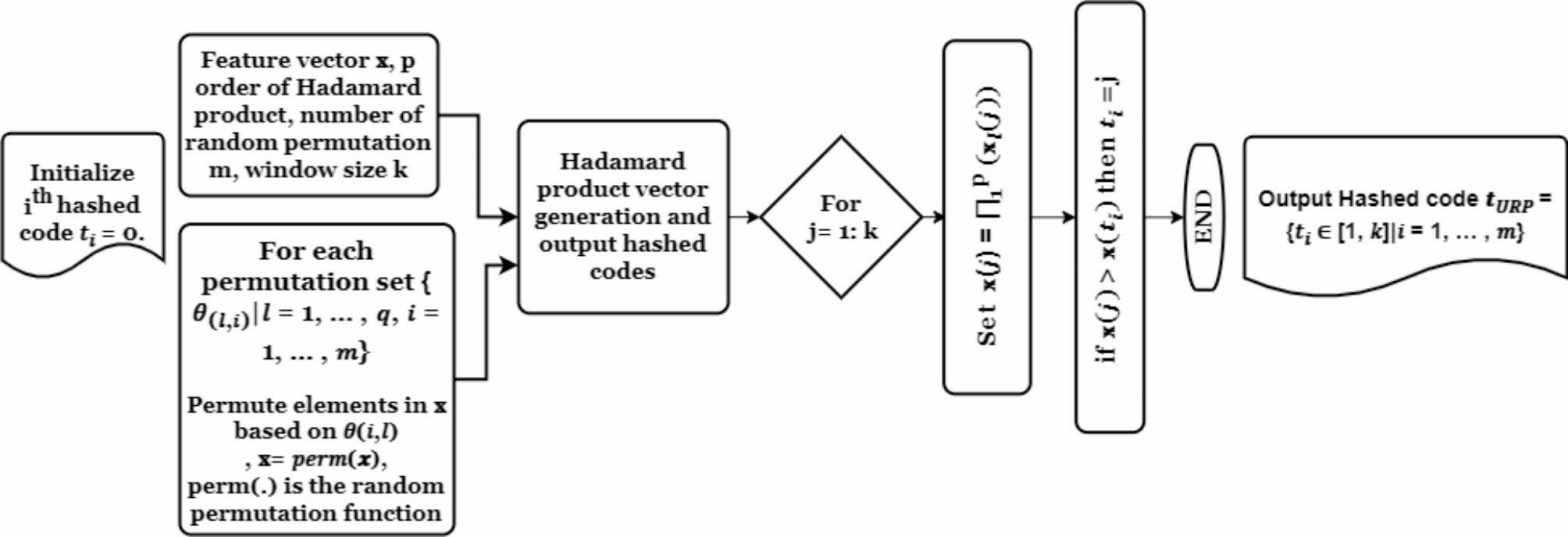
IoM-URP algorithm.

Another type of IoM hashing is IoM-URP. The IoM-URP hashing method relies on random permutation^46^. Weights are controlled through choosing the variable *k* of the inputs of the biometric vectors. IoM-URP algorithm is depicted in Fig. 6.

The hashing techniques chosen; Bio-Hashing, MLP Hashing, IoM-GRP, and IoM-URP present unique benefits compared to alternative hashing methods. These techniques enhance security through non-invertible transformations, ensuring that original biometric data remains unrecoverable in the event of a hash compromise. They also facilitate diversity and non-likability, enabling the generation of multiple distinct hashes from the same biometric input, thereby safeguarding against cross-matching across various systems. Furthermore, these methods are computationally efficient, adaptable to different biometric modalities, and resistant to collusion, rendering them suitable for robust and scalable biometric security solutions. By opting for these techniques, one can achieve a strong equilibrium of security, efficiency, adaptability, and privacy. Such advantages render them particularly appropriate for contemporary biometric systems that necessitate strong protection against emerging threats while ensuring performance and user convenience.

## The experimental results

In this section, we discuss the experimental results of the proposed CanDark approach. First, we adjust the CanDark model parameters before conducting the experiments. Some of these parameters are; ***(i) Weight decay*** (set to 5 × 10^− 4^); the best value ranges from zero to 0.1. ***(ii) Momentum*** (set to 0.9). ***(iii) #epochs***, we select different number of epochs (20, 30, 40, 50, and 60) for the evaluation of the CanDark model. ***(iv) Learning rate (LR)*** (set to 0.01 and 0.001).

We measure the classification performance in terms of accuracy, specificity, precision, recall, and f_score_. For all metrics, a number of statistical parameters are computed such as mean, standard error of median, mean, standard deviation, mode, skewness, variance, standard error of skewness, minimum, and maximum range. The mean is the average value of the results. The standard error of mean determines the discrepancy of the results compared with the mean. The Median is the midst value of the experimental results. The mode appears most frequently in experiments. The standard deviation and variance values give an indication about the closeness of measurements around the mean value. Lower values of standard deviation and variance are preferred to higher values. Skewness measures the symmetry or asymmetry in the data distribution. The distribution is considered highly skewed if (skewness < -1 or skewness > 1), moderately skewed if (-1 < skewness < -0.5 or 0.5 < skewness < 1), and approximately symmetric if (-0.5 < skewness < 0.5). The standard error of skewness is the ratio of skewness to standard error. The range is the difference between the maximum and the minimum values. Tables 4, 5, 6, and 7 illustrate the performance of the proposed framework using Bio-Hashing, Multi-Layer Perceptron (MLP) Hashing, IoM-GRP, and IoM-URP, respectively. From these results, it could be noticed that:

I. ***For Bio-Hashing method***, the maximum accuracy, specificity, precision, recall, and f_score_ are 97.67%, 98.86%, 98.77%, 98.81%, and 97.65%, respectively. The range of accuracy, specificity, precision, recall, and f_score_ are 6.39, 11.36, 10.86, 11.91, and 6.22, respectively. The skewness of accuracy is (0.321) (approximately symmetric), specificity is (-1.141) (highly skewed), precision is (-1.018) (highly skewed), recall is (-1.017) (highly skewed), f_score_ is (0.343) (approximately symmetric). The maximum accuracy is achieved using (#epochs = 60 and LR = 0.001).
II. ***For MLP Hashing method***, the maximum accuracy, specificity, precision, recall, and f_score_ are 96.51%, 97.73%, 97.37%, 97.62%, and 96.47%, respectively. The range of accuracy, specificity, precision, recall, and f_score_ are 3.49, 4.55, 4.51, 9.52, and 3.97, respectively. The skewness of accuracy is (0.005) (approximately symmetric), specificity is (0.663) (moderately skewed), precision is (0.69) (moderately skewed), recall is (-1.008) (highly skewed), f_score_ is (-0.034) (approximately symmetric). The maximum accuracy is achieved using (#epochs = 60 and LR = 0.01).
III. ***For IoM-GRP method***, the maximum accuracy, specificity, precision, recall, and f_score_ are 97.67%, 97.73%, 97.62%, 97.62%, and 97.62%, respectively. The range of accuracy, specificity, precision, recall, and f_score_ are 5.23, 4.55, 4.85, 5.95, and 5.4, respectively. The skewness of accuracy is (-1.179) (highly skewed), specificity is (-0.705) (moderately skewed), precision is (-0.769) (moderately skewed), recall is (-0.911) (moderately skewed), f_score_ is (-1.173) (highly skewed). The maximum accuracy is achieved using (#epochs = 50 and LR = 0.001).
IV. ***For IoM-URP method***, the maximum accuracy, specificity, precision, recall, and f_score_ are 98.81%, 98.73%, 99.82%, 98.94%, and 99.37%, respectively. The range of accuracy, specificity, precision, recall, and f_score_ are 2.01, 1.86, 3.08, 2.73, and 2.4, respectively. The skewness of accuracy is (-0.254) (approximately symmetric), specificity is (-0.747) (moderately skewed), precision is (-0.098) (approximately symmetric), recall is (-0.259) (approximately symmetric), f_score_ is (0.532) (moderately skewed). The maximum accuracy is achieved using (#epochs = 50 and LR = 0.001).
V. ***According to accuracy***, the mean value for IoM-URP (97.89%) > IoM-GRP (95.93%) > MLP Hashing (94.7%) > Bio-Hashing (94.29%). The variance value for IoM-URP (0.64) < MLP Hashing (2.516) < IoM-GRP (2.923) < Bio-Hashing (4.99). The standard deviation value for IoM-URP (0.799) < MLP Hashing (1.586) < IoM-GRP (1.7) < Bio-Hashing (2.23). The range for Bio-Hashing (6.39) > IoM-GRP (5.23) > MLP Hashing (3.49) > IoM-URP (2.01).
VI. ***According to specificity***, the mean value for IoM-URP (98%) > IoM-GRP (96.25%) > MLP Hashing (95.05%) > Bio-Hashing (94.77%). The variance value for IoM-URP (0.324) < IoM-GRP (2.23) < MLP Hashing (2.64) < Bio-Hashing (11.8). The standard deviation value for IoM-URP (0.569) < IoM-GRP (1.52) < MLP Hashing (1.62) < Bio-Hashing (3.44). The range for Bio-Hashing (11.36) > (IoM-GRP (4.55) and MLP Hashing (4.55)) > IoM-URP (1.86).
VII. ***According to precision***, the mean value for IoM-URP (98.24%) > IoM-GRP (96.05%) > Bio-Hashing (94.63%) > MLP Hashing (94.45%). The variance value for IoM-URP (0.915) < MLP Hashing (2.089) < IoM-GRP (2.577) < Bio-Hashing (10.67). The standard deviation value for IoM-URP (0.9566) < MLP Hashing (1.445) < IoM-GRP (1.605) < Bio-Hashing (3.266). The range for Bio-Hashing (10.86) > IoM-GRP (4.85) > MLP Hashing (4.51) > IoM-URP (3.08).
VIII. ***According to recall***, the mean value for IoM-URP (97.684%) > IoM-GRP (95.597%) > MLP Hashing (94.764%) > Bio-Hashing (93.811%). The variance value for IoM-URP (0.628) < IoM-GRP (5.051) < MLP Hashing (9.818) < Bio-Hashing (13.484). The standard deviation value for IoM-URP (0.7922) < IoM-GRP (2.247) < MLP Hashing (3.133) < Bio-Hashing (3.672). The range for Bio-Hashing (11.91) > MLP Hashing (9.52) > IoM-GRP (5.95) > IoM-URP (2.73).
IX. ***According to f***_***score***_, the mean value for IoM-URP (97.955%) > IoM-GRP (95.82%) > MLP Hashing (94.574%) > Bio-Hashing (94.14%). The variance value for IoM-URP (0.517) < MLP Hashing (2.856) < IoM-GRP (3.16) < Bio-Hashing (5.328). The standard deviation value for IoM-URP (0.719) < MLP Hashing (1.6899) < IoM-GRP (1.7775) < Bio-Hashing (2.308). The range for Bio-Hashing (6.22) > IoM-GRP (5.4) > MLP Hashing (3.97) > IoM-URP (2.4).
X. IoM-URP method is superior to all other cancelable techniques in terms of all performance metrics and statistical parameters.

**Fig. 7:**
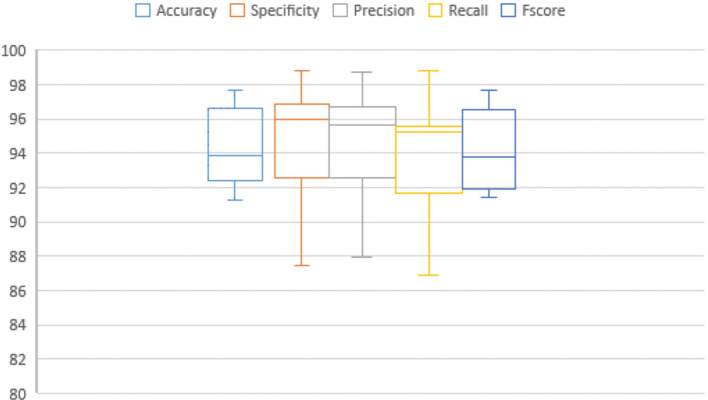
Graphical representation of the proposed framework using Bio-Hashing cancelable technique.

**Fig. 8:**
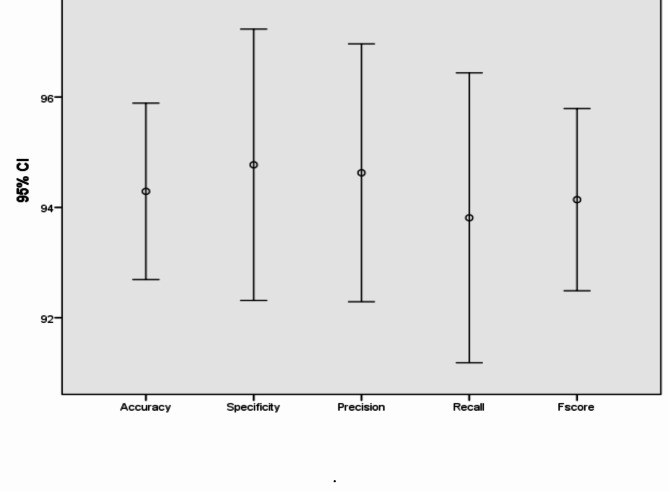
Error bar of accuracy, specificity, precision, recall, and f_score_ of the proposed framework using Bio-Hashing cancelable technique.

**Fig. 9:**
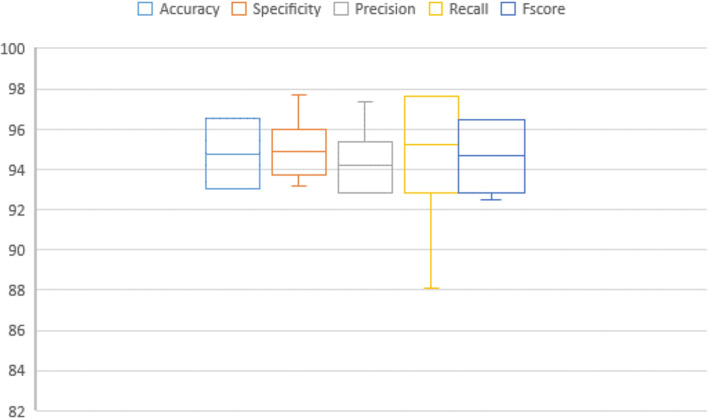
Graphical representation of the proposed framework using MLP Hashing cancelable technique.

**Fig. 10:**
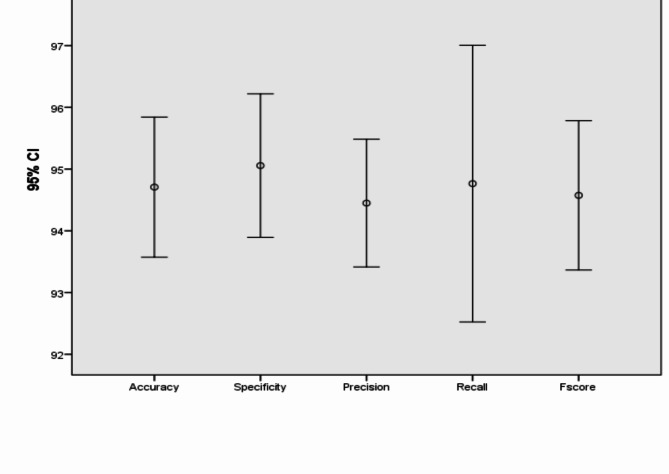
Error bar of accuracy, specificity, precision, recall, and f_score_ of the proposed framework using MLP Hashing cancelable technique.

**Fig. 11:**
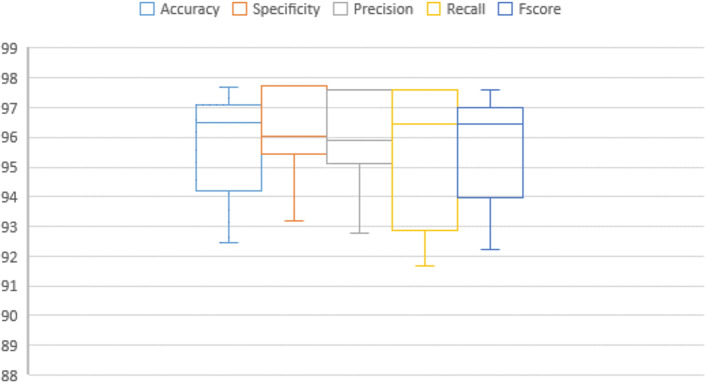
Graphical representation of the proposed framework using IoM-GRP cancelable technique.

**Fig. 12:**
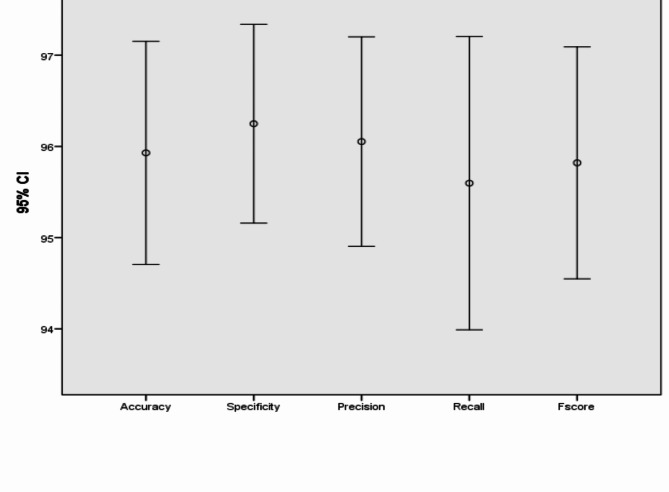
Error bar of accuracy, specificity, precision, recall, and f_score_ of the proposed framework using IoM-GRP cancelable technique.

**Fig. 13:**
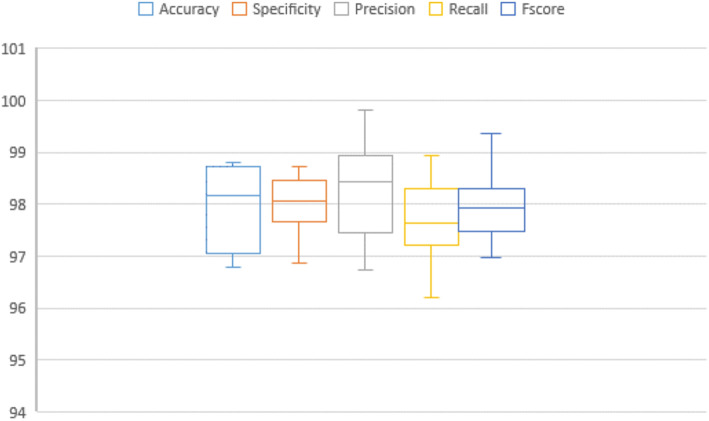
Graphical representation of the proposed framework using IoM-URP cancelable technique.

**Fig. 14:**
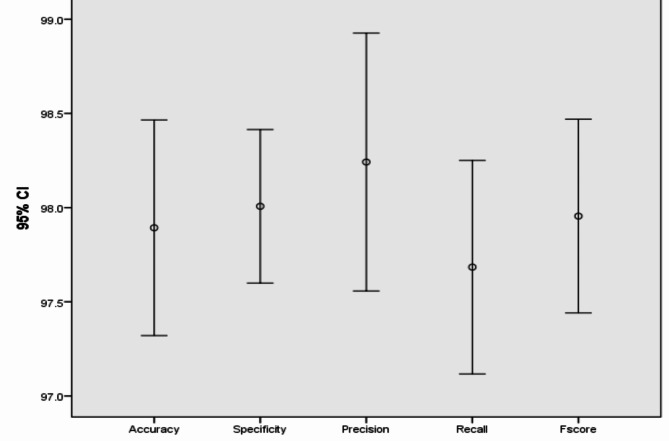
Error bar of accuracy, specificity, precision, recall, and f_score_ of the proposed framework using IoM-URP cancelable technique.

In addition, Figs. 7, 8, 9, 10, 11, 12, 13) display graphical representations of the proposed framework using Bio-Hashing, MLP Hashing, IoM-GRP, and IoM-URP, respectively. Figures 8, 10, 12, and 14 depict the error bars of accuracy, specificity, precision, recall, and f_score_ of the proposed framework using Bio-Hashing, MLP Hashing, IoM-GRP, and IoM-URP, respectively. Error bars indicate the estimated error and the uncertainty of experiments.

**Table 4:** Performance and numerical statistical analysis of the proposed framework using Bio-hashing cancelable technique.

#epochs	LR	Accuracy	Specificity	Precision	Recall	F_score_
20	0.01	92.44	90.91	90.80	94.05	92.40
	0.001	94.19	93.18	93.2	95.24	94.12
30	0.01	92.44	97.73	97.33	86.90	91.82
	0.001	97.09	98.86	98.77	95.24	96.97
40	0.01	95.24	95.45	95.24	95.7	95.24
	0.001	93.60	94.32	93.98	92.86	93.41
50	0.01	91.28	87.50	87.91	95.29	91.43
	0.001	96.51	96.22	96.43	96.43	96.43
60	0.01	92.44	96.13	96.10	88.10	91.93
	0.001	97.67	96.59	96.51	98.81	97.65
Mean		94.2900	94.7720	94.6270	93.811	94.140
Std. Error of Mean		0.70639	1.08716	1.03297	1.1612	0.72995
Median		93.8950	96.0200	95.6700	95.240	93.765
Mode		92.44	96.59	87.91^a^	95.24	91.43^a^
Std. Deviation		2.23381	3.43790	3.26652	3.6720	2.3083
Variance		4.990	11.819	10.670	13.484	5.328
Skewness		0.321	-1.141	-1.018	-1.017	0.343
Std. Error of Skewness		0.687	0.687	0.687	0.687	0.687
Range		6.39	11.36	10.86	11.91	6.22
Minimum		91.28	87.50	87.91	86.90	91.43
Maximum		97.67	98.86	98.77	98.81	97.65

**Table 5:** Performance and numerical statistical analysis of the proposed framework using MLP hashing cancelable technique.

#epochs	LR	Accuracy	Specificity	Precision	Recall	F_score_
20	0.01	93.6	93.11	92.8	92.82	92.86
	0.001	96.3	95.19	95.37	97.6	96.5
30	0.01	93.9	93.2	92.1	92.91	92.86
	0.001	96.3	95.4	95.33	97.68	96.47
40	0.01	94.77	94.32	94.12	95.24	94.67
	0.001	95.93	97.62	94.25	97.62	95.91
50	0.01	93.02	97.73	97.37	88.10	92.50
	0.001	93.02	93.18	92.86	92.86	92.86
60	0.01	96.51	95.45	95.35	97.62	96.47
	0.001	94.77	94.32	94.12	95.24	94.67
Mean		94.7080	95.0560	94.4490	94.764	94.574
Std. Error of Mean		0.50155	0.51362	0.45703	0.99087	0.53440
Median		94.7700	94.8850	94.1850	95.240	94.670
Mode		93.02	95.45	92.86^a^	97.62	92.86^a^
Std. Deviation		1.58605	1.62421	1.44524	3.1334	1.6899
Variance		2.516	2.638	2.089	9.818	2.856
Skewness		0.005	0.663	0.690	-1.008	− 0.034
Std. Error of Skewness		0.687	0.687	0.687	0.687	0.687
Range		3.49	4.55	4.51	9.52	3.97
Minimum		93.02	93.18	92.86	88.10	92.50
Maximum		96.51	97.73	97.37	97.62	96.47

**Table 6:** Performance and numerical statistical analysis of the proposed framework using IoM-GRP cancelable technique.

#epochs	LR	Accuracy	Specificity	Precision	Recall	F_score_
20	0.01	97.23	97.13	97.49	96.42	97.01
	0.001	96.51	95.1	95.31	97.63	96.43
30	0.01	94.19	95.45	95.12	92.86	93.92
	0.001	97.01	97.6	97.2	96.2	97.01
40	0.01	96.2	95.6	95.35	97.62	96.44
	0.001	94.7	95.7	95.25	92.13	93.96
50	0.01	96.51	96.59	96.43	96.43	96.41
	0.001	97.67	97.73	97.62	97.62	97.62
60	0.01	97.09	97.73	97.59	96.43	97.01
	0.001	92.44	93.18	92.77	91.67	92.22
Mean		95.9290	96.2490	96.0530	95.597	95.820
Std. Error of Mean		0.54067	0.48146	0.50763	0.71069	0.56211
Median		96.5100	96.0200	95.8900	96.430	96.470
Mode		96.51^a^	95.45^a^	97.59	96.43	97.01
Std. Deviation		1.70974	1.52250	1.60528	2.2473	1.7775
Variance		2.923	2.318	2.577	5.051	3.160
Skewness		-1.179	− 0.705	− 0.769	− 0.911	-1.173
Std. Error of Skewness		0.687	0.687	0.687	0.687	0.687
Range		5.23	4.55	4.85	5.95	5.40
Minimum		92.44	93.18	92.77	91.67	92.22
Maximum		97.67	97.73	97.62	97.62	97.62

**Table 7:** Performance and numerical statistical analysis of the proposed framework using IoM-URP cancelable technique.

#epochs	LR	Accuracy	Specificity	Precision	Recall	F_score_
20	0.01	98.23	98.42	97.05	98.27	97.65
	0.001	97.06	98.53	97.6	98.11	97.85
30	0.01	97.06	97.95	98.19	97.64	97.91
	0.001	96.8	96.87	96.74	97.26	96.99
40	0.01	98.72	97.77	98.68	97.28	97.97
	0.001	97.1	97.46	97.75	96.21	96.97
50	0.01	98.72	98.44	99.02	98.45	98.73
	0.001	98.81	98.73	98.9	97.02	97.95
60	0.01	98.12	97.72	98.67	97.66	98.16
	0.001	98.31	98.18	99.82	98.94	99.37
Mean		97.8930	98.0070	98.2420	97.684	97.955
Std. Error of Mean		0.25296	0.18006	0.30253	0.25052	0.22737
Median		98.1750	98.0650	98.4300	97.650	97.930
Mode		97.06^a^	96.87^a^	96.74^a^	96.21^a^	96.97^a^
Std. Deviation		0.79993	0.56941	0.95668	0.79220	0.71900
Variance		0.640	0.324	0.915	0.628	0.517
Skewness		− 0.254	− 0.747	− 0.098	− 0.259	0.532
Std. Error of Skewness		0.687	0.687	0.687	0.687	0.687
Range		2.01	1.86	3.08	2.73	2.40
Minimum		96.80	96.87	96.74	96.21	96.97
Maximum		98.81	98.73	99.82	98.94	99.37

**Fig. 15:**
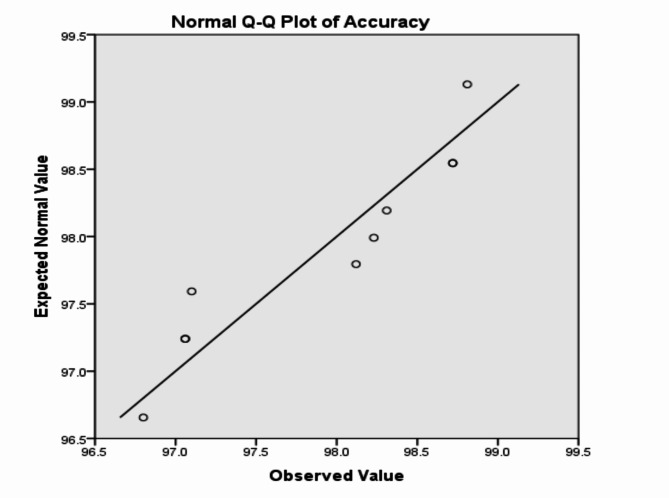
Normal Q-Q plot of the accuracy of the proposed framework using IoM-URP cancelable technique.

**Fig. 16:**
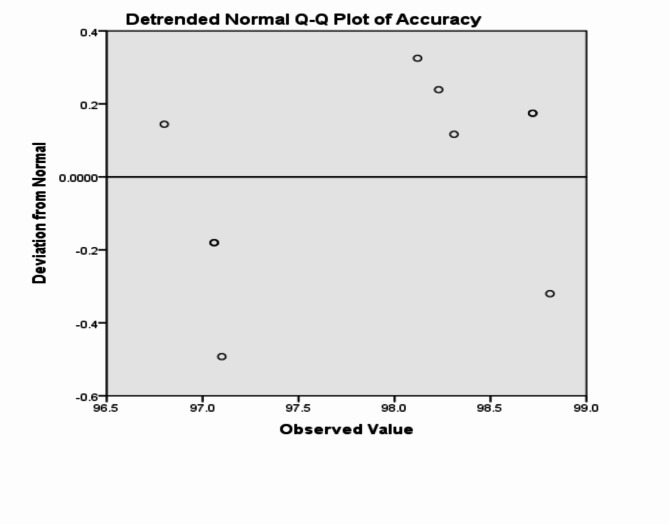
Detrended normal Q-Q plot of the accuracy of the proposed framework using IoM-URP cancelable technique.

**Fig. 17:**
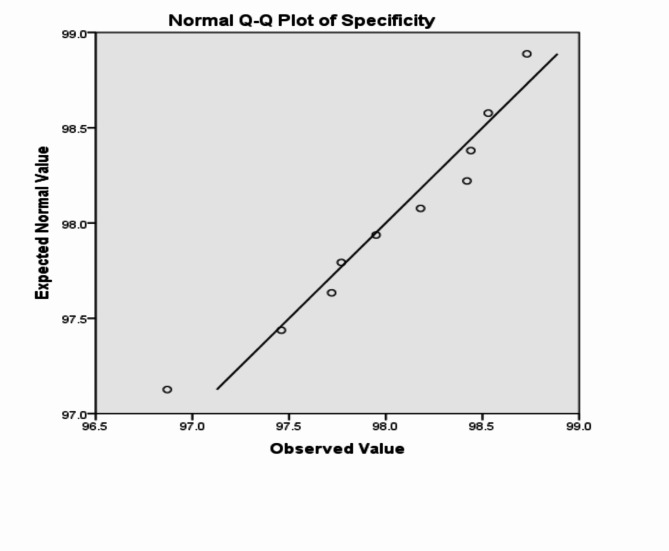
Normal Q-Q plot of the specificity of the proposed framework using IoM-URP cancelable technique.

**Fig. 18:**
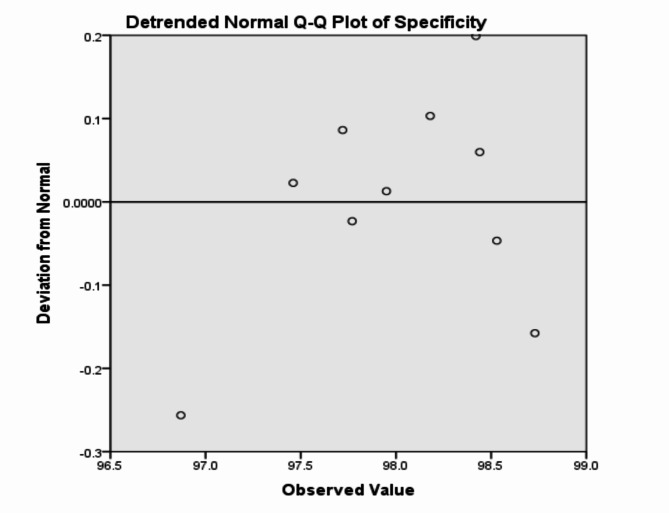
Detrended normal Q-Q plot of the specificity of the proposed framework using IoM-URP cancelable technique.

**Fig. 19:**
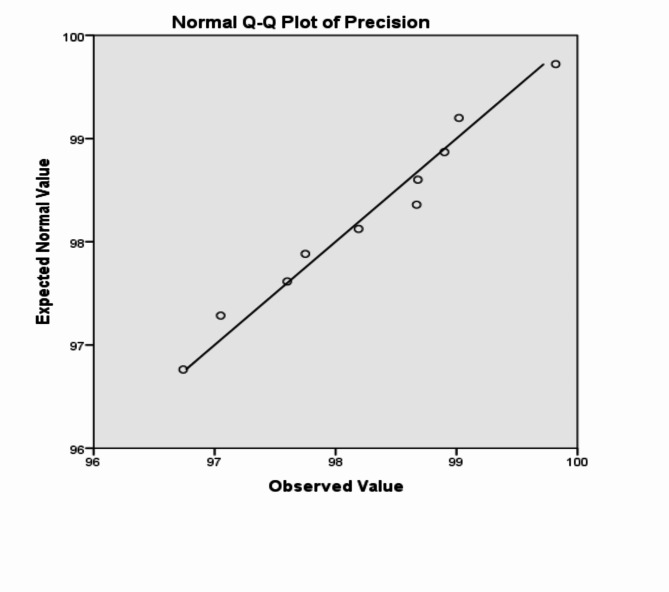
Normal Q-Q plot of the precision of the proposed framework using IoM-URP cancelable technique.

**Fig. 20:**
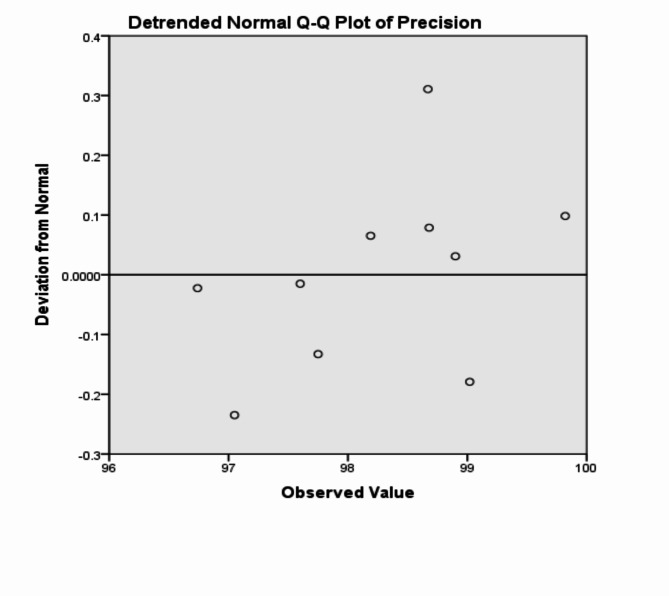
Detrended normal Q-Q plot of the precision of the proposed framework using IoM-URP cancelable technique.

**Fig. 21:**
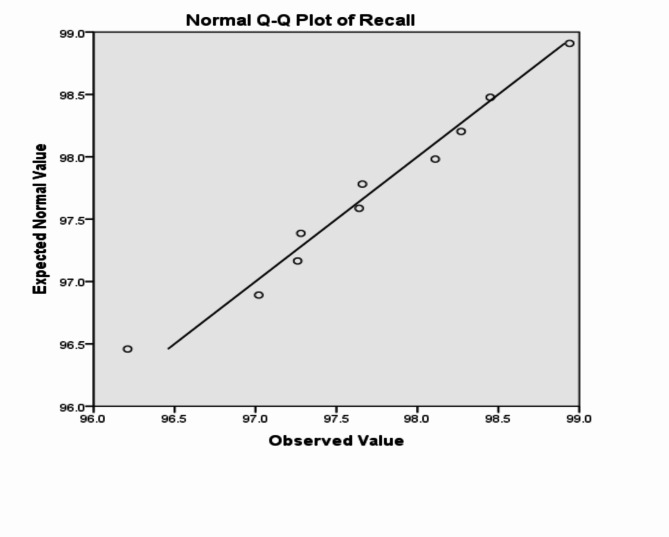
Normal Q-Q plot of the recall of the proposed framework using IoM-URP cancelable technique.

**Fig. 22:**
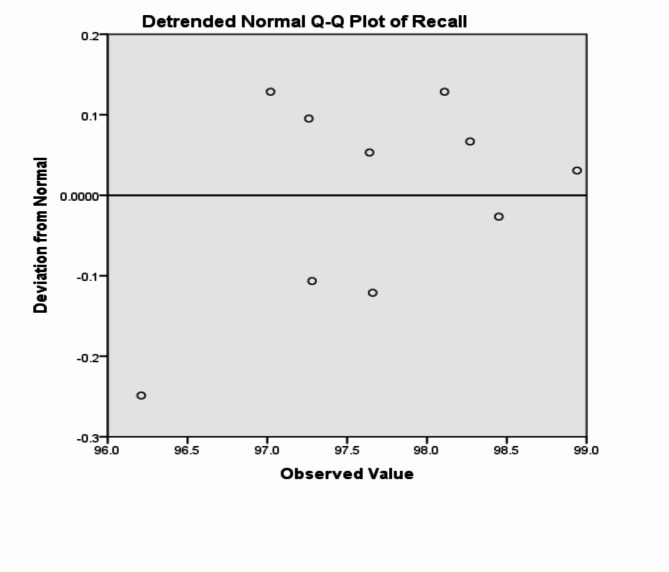
Detrended normal Q-Q plot of the recall of the proposed framework using IoM-URP cancelable technique.

**Fig. 23:**
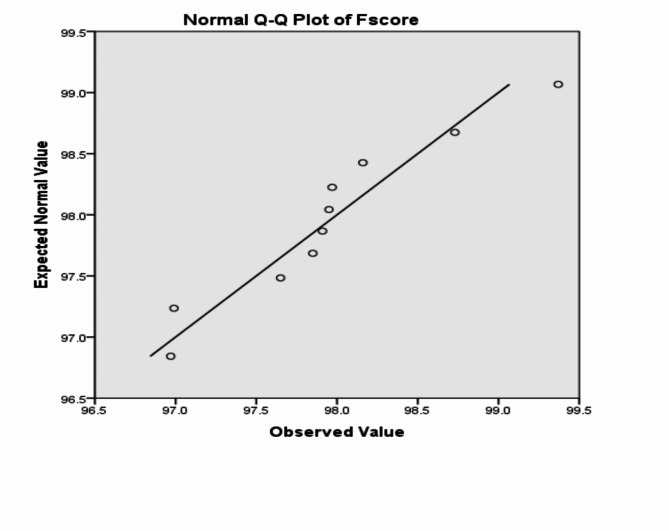
Normal Q-Q plot of the f_score_ of the proposed framework using IoM-URP cancelable technique.

**Fig. 24:**
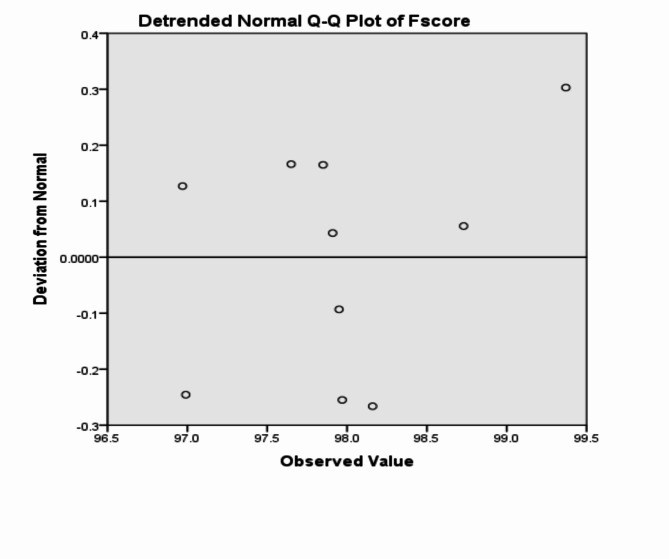
Detrended normal Q-Q plot of the f_score_ of the proposed framework using IoM-URP cancelable technique.

The analysis of the previous measurements confirms the superiority of using IoM-URP cancelable algorithm to provide cancelability of the extracted features from the DarkNet-53 CNN model. For further analysis of the proposed framework using IoM-URP method, Figs. 15, 16, 17, 18, 19, 20, 21, 22, and 23 display the normal Q-Q plot of the accuracy, specificity, precision, recall, and f_score_, respectively. The Q-Q plot determines if the measurements are normally distributed (points fall on the 45-degree line) or not. Moreover, Figs. 16, 18, 20, 22, 24 present the detrended normal Q-Q plot of the accuracy, specificity, precision, recall, and f_score_, respectively. These plots are used to find the difference between the observed and the predicted measurements. The results are considered to be normally distributed if that difference lies around the zero line.

**Fig. 25:**
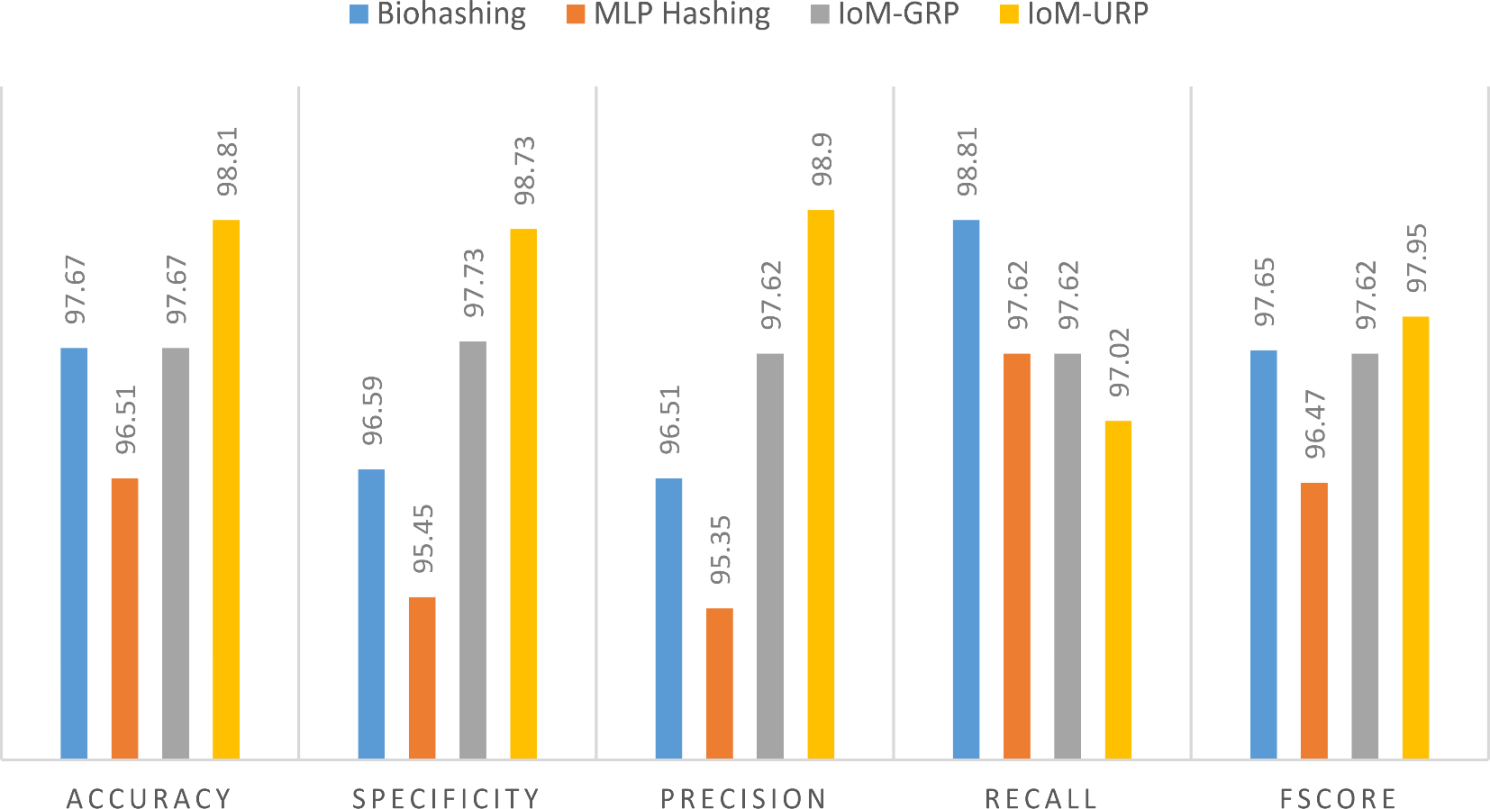
Graphical comparison between accuracy, specificity, precision, recall, and f_score_ of the proposed framework under various cancelable techniques.

Besides, Table 8; Fig. 25 proves the superiority of using IoM-URP over other cancelable methods through a numerical and graphical comparisons between the accuracy, specificity, precision, recall, and f_score_. Finally, Table 9 provides the performance of the proposed CanDark model against state-of-the-art techniques.

**Table 8:** Performance of the proposed framework under various cancelable techniques.

Cancelable technique	Accuracy	Specificity	Precision	Recall	F_score_
Bio-hashing	97.67	96.59	96.51	98.81	97.65
MLP Hashing	96.51	95.45	95.35	97.62	96.47
IoM-GRP	97.67	97.73	97.62	97.62	97.62
IoM-URP	98.81	98.73	98.9	97.02	97.95

**Table 9:** Performance of the proposed CanDark model against state-of-the-art techniques.

Method			Accuracy	Precision	Recall	F_score_
Irmak^31^			91.38	90	86	88
Akin^33^			98.25	100	96	98
Sitaula^34^			87.13	85	85	85
Altun^36^			96	99	97	98
Ahsan^39^	Batch Size = 10 Epochs = 50 LR = 0.001	VGG16	81	86	81	80
		InceptionV2	90	88	88	77
		ResNet-50	56	32	56	40
		ResNet-101	56	32	56	40
		MobileNetV2	81	82	81	81
		VGG19	93	94	94	94
	Batch Size = 10 Epochs = 50 LR = 0.001	VGG16	93	93	90	91
		InceptionV2	98	98	97	98
		ResNet-50	72	51	72	60
		ResNet-101	72	36	50	42
		MobileNetV2	99	99	99	99
		VGG19	90	89	86	87
Proposed CanDark Model			98.81	98.9	97.02	97.95

The proposed method outperforms all other methods across most criteria, with the exception of Ahsan^39^ (using MobileNetV2). This discrepancy arises because Ahsan^39^ employs an 80% training rate, while our proposed method uses a 70% training rate. However, we also tested our CanDark model using an 80% training rate, and the results were as follows: accuracy = 99.38%, Precision = 99.53%, recall = 99.16% and f_score_ = 99.34%.

## Conclusion

As COVID-19 virus infections drop globally, the Monkeypox virus is steadily emerging. Early detection of them may be achievable with the help of AI-based detection approaches based on deep learning. This study used 770 skin pictures, which were split into four categories: measles, chickenpox, Monkeypox, and normal cases. The proposed CanDark model is a complete paradigm that uses cancellable biometrics to identify cases of Monkeypox. It relies on using the DarkNet-53 CNN model to extract features from images of Monkeypox skin. Then, cancellable biometric techniques including bio-hashing, MLP, IoM-GRP, and IoM-URP hashing techniques can be used to protect the output information. The proposed framework achieved an accuracy of 98.81%, a specificity of 98.73%, a precision of 98.9%, a recall of 97.02%, and f_score_ of 97.95%. We can enlarge the data set and evaluate the suggested model’s performance in subsequent work. Moreover, we can test the effectiveness of Monkeypox case detection and classification using various pre-trained models and a scratch CNN models.

## Data Availability

Data set available on: https://www.kaggle.com/datasets/dipuiucse/monkeypoxskinimagedataset, and used by (bala et al., 2023monkeynet) MonkeyNet: A robust deep convolutional neural network for monkeypox disease detection and classification. 10.1016/j.neunet.2023.02.022.

## References

[1] Antunes, F., Cordeiro, R. & Virgolino, A. Monkeypox: From a neglected tropical disease to a public health threat. *Infect. Dis. Rep.* **14**(5), 772–783 (2022). 36286200 10.3390/idr14050079 PMC9602669

[2] Control P. ASSESSMENT and Monkeypox multi-country outbreak (2022).

[3] Nakazawa, Y. et al. Phylogenetic and ecologic perspectives of a monkeypox outbreak, southern Sudan, 2005. *Emerg Infect Dis.* **19**(2) (2013). 10.3201/eid1902.121220 PMC3559062 23347770

[4] Ladnyj, I., Ziegler, P. & Kima, E. A human infection caused by monkeypox virus in Basankusu Territory, Democratic Republic of the Congo. *Bull. World Health Organ.* **46**(5) (1972). PMC2480792 4340218

[5] Marennikova, S., Šeluhina, E., Ceva, N., Čimiškjan, K. & Macevič, G. Isolation and properties of the causal agent of a new variola-like disease (monkeypox) in man. *Bull. World Health Organ.* **46**(5) (1972). PMC2480798 4340219

[6] Yinka-Ogunleye, A. et al. Outbreak of human monkeypox in Nigeria in 2017–18: A clinical and epidemiological report. *Emerg. Infect. Dis.* **19**(8), 872–879 (2019). 10.1016/S1473-3099(19)30294-4 PMC9628943 31285143

[7] Lourie, B. et al. Human infection with monkeypox virus: Laboratory investigation of six cases in West Africa. *Emerg. Infect. Dis.* **46**(5) (1972). PMC2480791 4340223

[8] Tiee, M., Harrigan, R., Thomassen, H. & Smith, T. Ghosts of infections past: Using archival samples to understand a century of monkeypox virus prevalence among host communities across space and time. *Bull. World Health Organ.* **5**(1) (2018). 10.1098/rsos.171089 PMC5792900 29410823

[9] Magnus, P., Andersen, E., Petersen, K. & Birch-Andersen, A. A pox‐like disease in cynomolgus monkeys. *Acta Pathol. Microbiol. Scand.* **46**(2), 156–176 (1959).

[10] Parker, S. & Buller, R. A review of experimental and natural infections of animals with monkeypox virus between 1958 and 2012. *Future Virol.* **8**(2), 129–157 (2013). 23626656 10.2217/fvl.12.130 PMC3635111

[11] Simpson, K. et al. Human monkeypox–after 40 years, an unintended consequence of smallpox eradication. *Vaccine* **38**(33), 5077–5081 (2020). 32417140 10.1016/j.vaccine.2020.04.062 PMC9533855

[12] Fenner, F. and p. sciences, Smallpox: Emergence, global spread, and eradication. *Hist. Philos. Life Sci.* 397–420 (1993). 7529932

[13] Alakunle, E., Moens, U., Nchinda, G. & Okeke, M. Monkeypox virus in Nigeria: Infection biology, epidemiology, and evolution. *Viruses* **12**(11) (2020). 10.3390/v12111257 PMC7694534 33167496

[14] Vaughan, A. et al. Two cases of monkeypox imported to the United Kingdom, September 2018. *Euro Surveill* **23**(38) (2018). 10.2807/1560-7917.ES.2018.23.38.1800509 PMC6157091 30255836

[15] Yong, S. et al. Imported Monkeypox, Singapore. *Travel Med. Infect. Dis.* **26**, 8 (2020). 10.3201/eid2608.191387 PMC7392406 32338590

[16] Costello, V. et al. Imported monkeypox from international traveler, Maryland, USA, 2021. *Emerg. Infect. Dis.* **28**, 5 (2022). 10.3201/eid2805.220292 PMC9045429 35263559

[17] Guarner, J. et al. Monkeypox transmission and pathogenesis in prairie dogs. *Emerg. Infect. Dis.* **10**, 3 (2004). 10.3201/eid1003.030878 PMC3322777 15109408

[18] Russo, A. T. et al. An overview of tecovirimat for smallpox treatment and expanded anti-orthopoxvirus applications. *CDC* **19**(3), 331–344 (2021). 10.1080/14787210.2020.1819791 PMC9491074 32882158

[19] https://www.cdc.gov/poxvirus/mpox/response/2022/world-map.html, october (2023).

[20] Reynolds, M. G. et al. Spectrum of infection and risk factors for human monkeypox, United States. *Emerg. Infect. Dis.* **13**, 9 (2007). 10.3201/eid1309.070175 PMC2857287 18252104

[21] Saad, W., Shalaby, W., Shokair, M., Sayed, F. & Abdellatef, E. COVID-19 classification using deep feature concatenation technique. *J. Ambient Intell. Hum. Comput.* **13**, 2025–2043 (2022). 10.1007/s12652-021-02967-7 PMC7924021 33680212

[22] Ravì, D. et al. Deep learning for health informatics. *IEEE J. Biomed. Health Inf.* **21**(1), 4–21 (2016). 10.1109/JBHI.2016.2636665 28055930

[23] Ciregan, D., Meier, U. & Schmidhuber, J. Multi-column deep neural networks for image classification. In *IEEE Conference on Computer Vision and Pattern Recognition*, 3642–3649 (2012).

[24] Özkaya, U., Öztürk, Ş., Budak, S., Melgani, F. & Polat, K. Classification of COVID-19 in chest CT images using convolutional support vector machines. *Electr. Eng. Syst. Sci.* (2020).

[25] Cohen, J. P. et al. Predicting covid-19 pneumonia severity on chest x-ray with deep learning. *Cureus* **12**(7) (2020). 10.7759/cureus.9448 PMC7451075 32864270

[26] Khan, G. & Ullah, I. Efficient technique for monkeypox skin disease classification with clinical data using pre-trained models. *J. Innov. Image Process.* **5**(2), 192–213 (2023).

[27] Özkan, İ. & M. ÇELİK and Detection of monkeypox among different pox diseases with different pre-trained deep learning models. *J. Inst. Sci. Technol.* **13**(1), 10–21 (2023).

[28] Pal, M. et al. Deep and transfer learning approaches for automated early detection of monkeypox (mpox) alongside other similar skin lesions and their classification. *ACS Omega* **23**(35), 31747–31757 (2023). 10.1021/acsomega.3c02784 PMC10483519 37692219

[29] Pal, M. et al. COVID-19 prognosis from chest X-ray images by using deep learning approaches: A next generation diagnostic tool. *J. Pure Appl. Microbiol.* **17**(2), 919–930 (2023).

[30] Sahin, V., Oztel, I. & Oztel, G. Human monkeypox classification from skin lesion images with deep pre-trained network using mobile application. *J. Med. Syst.* **46**, 11 (2022). 10.1007/s10916-022-01863-7 PMC9548428 36210365

[31] Irmak, M., Aydin, T. & Yağanoğlu, M. Monkeypox skin lesion detection with MobileNetV2 and VGGNet models. In *Medical Technologies Congress (TIPTEKNO)*, 1–4 (2022).

[32] Alcalá-Rmz, V., Villagrana-Bañuelos, K., Celaya-Padilla, J., Galván-Tejada, J. & Gamboa-Rosales, H. & Galván-Tejada, C. Convolutional neural network for monkeypox detection. In *International Conference on Ubiquitous Computing and Ambient Intelligence*, 89–100 (2022).

[33] Akin, K., Gurkan, C., Budak, A. & Karataş, H. Classification of monkeypox skin lesion using the explainable artificial intelligence assisted convolutional neural networks. *Eur. J. Sci. Technol.* **40**, 106–110 (2022).

[34] Sitaula, C. & Shahi, T. Monkeypox virus detection using pre-trained deep learning-based approaches. *J. Med. Syst.* **46**, 11 (2022). 10.1007/s10916-022-01868-2 PMC9535233 36201085

[35] Ali, S. N. et al. Monkeypox skin lesion detection using deep learning models: A feasibility study. *Comput. Vis. Pattern Recognit.* (2022).

[36] Altun, M. et al. Monkeypox detection using CNN with transfer learning. *Sensors* **23**, 4 (2023). 10.3390/s23041783 PMC9964526 36850381

[37] Islam, T., Hussain, M., Chowdhury, F. & Islam, B. Can artificial intelligence detect monkeypox from digital skin images? *Bioexiv* (2022).

[38] Haque, M., Ahmed, M., Nila, R. & Islam, S. Classification of human monkeypox disease using deep learning models and attention mechanisms. *Image Video Process.* (2022).

[39] Ahsan, M. M. et al. Transfer learning and local interpretable model agnostic based visual approach in Monkeypox Disease Detection and classification: A deep learning insights. *Image Video Process.* (2022).

[40] https://www.kaggle.com/datasets/dipuiucse/monkeypoxskinimagedataset.

[41] Rathgeb, C., Breitinger, F. & Busch, C. Alignment-free cancelable iris biometric templates based on adaptive bloom filters. In *International Conference on Biometrics (ICB)*, 1–8 (2013).

[42] Manisha & Kumar, N. Cancelable biometrics: A comprehensive survey. *Artif. Intell. Rev.* **53**, 3403–3446 (2020).

[43] Shahreza, H., Hahn, V. & Marcel, S. Mlp-hash: Protecting face templates via hashing of randomized multi-layer perceptron. *Comput. Vis. Pattern Recognit.* (2022).

[44] Tan, F., Ong, T., Tee, C. & Teoh, A. Image hashing enabled technique for biometric template protection. *TENCON IEEE Region 10 Conference*, 1–5 (2009).

[45] Yee, L. & De Silva, L. Application of multilayer perceptron network as a one-way hash function. In *Proceedings of the 2002 International Joint Conference on Neural Networks. IJCNN’02*, Vol. 2, No. 2, 1459–1462 (2002).

[46] Jin, Z., Hwang, J., Lai, Y., Kim, S. & Teoh, A. Ranking-based locality sensitive hashing-enabled cancelable biometrics: Index-of-max hashing. *IEEE Trans. Inform. Forensics Secur.*. **13**(2), 393–407 (2017).

